# Nuances in the Management of Aggressive Lymphomas

**Published:** 2017-04-01

**Authors:** Paul A. Hamlin, Michelle Wisniewski

**Affiliations:** Memorial Sloan Kettering Cancer Center, New York New York

## Abstract

Treatment for both Hodgkin and Non-Hodgkin lymphoma now includes chemotherapy, targeted therapy, and new immunotherapies. As research continues to shine the light on tumor genetics, clinical variability, and new treatment approaches, advanced practitioners need to translate this information in the management of aggressive lymphomas.

Aggressive lymphomas are a heterogeneous population of tumors whose management is changing as their biology becomes better understood. Nuances in managing these patients, including current standards and future strategies, were discussed at 2016 JADPRO Live by Paul A. Hamlin, MD, and Michelle Wisniewski, MS, PA-C, of Memorial Sloan Kettering Cancer Center, New York. The two clinicians share patient care, alternating office visits.

Non-Hodgkin lymphoma is a common hematologic malignancy, with diffuse large B-cell lymphoma (DLBCL), an aggressive form, accounting for 31% of these tumors (Armitage & Weisenburger, 1998). At the conference, the speakers focused on DLBCL, which is now differentiated based on its cell of origin: germinal center B-cell (GCB) and activated B-cell (ABC) types. They also discussed DLBCL with *MYC* and *BCL2* and/or *BCL6* rearrangements, the so-called double-expressing and double-hit phenotypes, which are associated with a poor prognosis.

Dr. Hamlin noted that better understanding of the genetic basis of lymphoma is helping to "tease out the heterogeneity" of this malignancy. "These distinctions, according to biology, have implications for outcomes," he said.

## DIAGNOSIS: FIRST CLASSIFY BY CELL OF ORIGIN

The classification of DLBCL is still based on immunohistochemistry, although this test is "imperfect" and will eventually be replaced, according to Dr. Hamlin. "Going forward, we will likely use NanoString technology to rapidly distinguish between GBC and non-GBC/ABC biology or gene-expression profiling," he revealed.

Immunohistochemistry, however, currently remains a first step, added Ms. Wisniewski, who shared how she works closely with pathologists to make the diagnosis. "When we get outside pathology results, sometimes even before we see the patient, we submit these results to our own pathologists for confirmation," she explained. It is important to obtain cMYC status and to identify genetic abnormalities by fluorescence in situ hybridization (FISH), so these tests are requested as needed, added Dr. Hamlin.

**Figure 1:**
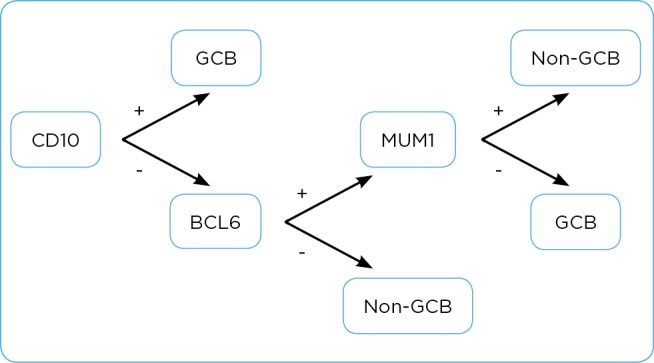
Decision tree for classification of diffuse large B-cell lymphoma based on immunohistochemistry. GCB = germinal center B cell. Information from Hans et al.(2004)

B-cell ontogeny defines the lymphoma biology. The simple "decision tree" by Hans et al. (2004) can help separate the two cell-of-origin types (Figure 1), he suggested. In this model, if patients are CD10-positive, they automatically have the GCB subtype. If they are CD10-negative, patients then must have BCL6 and MUM1 status determined. Patients who are CD10-negative and BCL6-negative are non-GC. Those who are CD10 -negative but BCL6-positive are further distinguished by their MUM1 status, with MUM1-negative patients GCB and MUM1-positive patients non-GCB.

"In short, CD10 and BCL6 are markers of GCB. The MUM1 is an activated B-cell marker," explained Ms. Wisniewski. With genetic information having become critical to treatment decisions, excisional biopsies are required for tissue acquisition, and fine-needle aspirations are no longer acceptable, Dr. Hamlin added. The majority of tumors will be fluorodeoxyglucose (FDG)-avid. Positron emission tomography–computed tomography (PET-CT) is standard imaging for FDG-avid lymphomas, whereas CT is indicated for nonavid histologies. If PET-CT is performed, a bone marrow biopsy is needed only if the PET result is negative and if identifying a discordant histology is important for patient management. PET is performed at baseline and at the end of treatment (or interim).

## PREDICTING OUTCOMES

The International Prognostic Index (IPI) still remains important for predicting a patient’s course. The index is based on age, performance status, lactate dehydrogenase (LDH) level, extranodal sites of disease, and disease stage. Revised IPI criteria for DLBCL in the era of rituximab (Rituxan) indicate there is no risk group with 4-year overall survival of less than 50%. In the population considered at lowest risk for relapse, 4-year survival is now 94%, dropping to 55% for the highest risk patients (Sehn et al., 2007).

Outcomes are also predictable based on the cell of origin and genetic makeup, and clinicians should be incorporating these factors, Dr. Hamlin advised. Germinal center B-cell tumors are associated with more than a doubling in survival over non-GCB tumors, even in the era of rituximab plus cyclophosphamide, doxorubicin, vincristine, and prednisone (R-CHOP).

"Large cell lymphomas are heterogeneous," he said. "We are starting to be able to say that DLBCL is not one disease but is made up of multiple genetic entities."

With this recognition, clinicians are incorporating mutational analysis into prognostication and treatment decision-making. "At our center, we are now doing gene sequencing on all patients to identify abnormalities that are actionable or predictive…. Clinically, we know there will be different outcomes…. In the near future, treatments will be based on those patterns," he predicted.

With the identification of recurrent mutations that could serve as therapeutic targets, along with the emergence of novel agents, there is some indication that this poor prognosis might be ameliorated.

Along with the cell of origin, clinicians need to determine a patient’s risk for secondary central nervous system (CNS) relapse (to guide treatment) and the presence of double-expressing or double-hit lymphoma.

A recently published validated prognostic model, based on almost 5,000 patients with aggressive B-cell lymphoma, includes six factors associated with an elevated risk of CNS relapse (Savage et al., 2014; Schmitz et al., 2016). The model, primarily based on the IPI factors plus adrenal or kidney involvement, can identify patients with more than a 10% risk of CNS involvement and can help select patients for prophylaxis, although this is not yet an exact science. "How we decrease that risk remains a question," Dr. Hamlin added. His practice is to give two cycles of high-dose methotrexate at the end of therapy or intrathecal therapy concurrent with treatment.

## CHALLENGING DOUBLE-HIT LYMPHOMAS

In addition to molecular subtype, the relevance of two proteins, MYC and BCL2, has recently been elucidated. Patients with double-hit lymphoma (~10% of DLBCL, most with a GCB cell of origin) have tumors that exhibit mutations on both these significant genes. They often respond poorly to the standard R-CHOP, with median survival of less than 1 year (Sarkozy, 2015). More patients (~30%), however, overexpress the MYC and BCL2 proteins. The clinical course of these double expressers is worse than that of standard patients with DLBCL but slightly better than the survival outcomes for patients with double-hit lymphoma.

When MYC and BCL2 are either both translocated or overexpressed, "they conspire to create an aggressive biology," Dr. Hamlin summed up.

Due to their markedly poor outcomes, alternative therapies are needed for patients with double-hit and double-expressing lymphoma. "With R-CHOP, our outcomes are woefully inadequate. We are prospectively trying to prove that one regimen may be better than another," Dr. Hamlin said. His own group is evaluating upfront transplant and treatment with dose-adjusted (DA) R-EPOCH (etoposide, doxorubicin, and cyclophosphamide with vincristine, prednisone, and rituximab). Early prospective outcomes appear promising.

## TREATING THE FRAIL AND THE ELDERLY

Many patients with DLBCL are in their 70s and 80s and have multiple comorbidities, often resulting in undertreatment. In a study led by Dr. Hamlin (Hamlin et al., 2014), information from the Surveillance, Epidemiology, and End Results (SEER) database of 9,333 DLBCL patients revealed that one-quarter of these older patients had not received treatment, and three-quarters received treatment that was often not of curative intent. "This occurred, even though they had curable disease. Were they so sick and frail that this was a life choice, or was there a lack of understanding?" he questioned.

Whether to treat for curative potential is a critical question in managing older patients. "How do we approach these patients in a thoughtful way," he asked, "so that we offer the best chance for good long-term outcomes but don’t expose them to toxic therapy that will not benefit them?"

**Figure 2:**
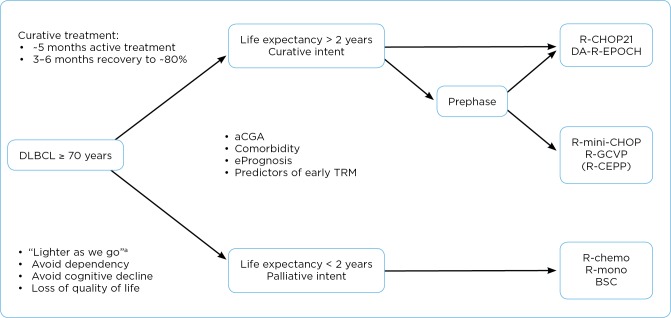
Algorithm for decision-making for patients with diffuse large B-cell lymphoma. aGreenstein and Holland (2014). ^a^CGA = abbreviated comprehensive geriatric assessment; TRM = transplant-related mortality; R-CHOP21 = rituximab, cyclophosphamide, doxorubicin, vincristine, and prednisone every 3 weeks; DA-R-EPOCH = dose-adjusted rituximab, etoposide, prednisone, vincristine, cyclophosphamide, and doxorubicin; R-mini-CHOP = attenuated CHOP-regimen with reduced cyclophosphamide and doxorubicin doses; R-GCVP = rituximab, gemcitabine, cyclophosphamide, vincristine, and prednisolone; R-CEPP = rituximab, cyclophosphamide, etoposide, procarbazine, and prednisolone; R-chemo = rituximab plus chemotherapy; R-mono = rituximab monotherapy; BSC = best supportive care."

One way to approach this question is to determine the patient’s life expectancy without DLBCL. For example, a 79-year-old man in average health is expected to live more than 5 years; therefore, his lymphoma is life-limiting, and curative intent may be in order. Palliative intent would be appropriate for an older, frailer person, with less than 2 years’ life expectancy (Figure 2).

Clinicians should remember that the elderly often value "quality" over "quantity" of life and may have an aversion to toxicity. "We need to integrate this into our thought process," indicated Dr. Hamlin. A tool called ePrognosis uses multiple factors to provide a prognosis and may be helpful in selecting treatment.

## TREATMENT APPROACHES

Since patients with large cell lymphoma respond well to both chemotherapy and monoclonal antibodies, most any type of treatment can ameliorate symptoms and possibly impact longevity. Standard treatment remains R-CHOP21 for six cycles, without maintenance, based on a lack of survival benefit (unlike in indolent lymphomas). Upfront transplant also has not proved to be beneficial in randomized trials.

"There is convincing evidence that transplant adds toxicity without benefiting most patients," Dr. Hamlin maintained. "Although the exception may be the 10% of patients with double-hit lymphoma—and this is still an open question."

Patients with a poor performance status or poor nutritional status at presentation are at risk for early treatment-related mortality. The emerging concept of "prephase" is a strategy for possibly reducing this risk by boosting physiologic reserves at treatment initiation (when toxicity is usually the worst).

His recommendation for this approach is a single 1-mg dose of vincristine plus prednisone at 100 mg for 7 days, as was used in the German High-Grade Non-Hodgkin’s Lymphoma Study Group (DSHNHL) NHL-B2 trial (Pfreundschuh et al., 2004) where the prephase protocol resulted in a 50% reduction in treatment-related mortality in cycles 1 and 2. A prospective study from France (Peyrade et al., 2014) validated this strategy as a "useful clinical maneuver," he added.

Since vincristine is associated with neuropathy and ileus, Dr. Hamlin and his team prefer a different prephase approach. They give one dose of rituximab (375 mg/m²) between days 1 and 14, plus 100 mg of prednisone for 5 to 10 days, prior to R-CHOP (or a similar regimen). They are evaluating their protocol in a pilot study of patients that are ≥ 70 years of age and also assessing inflammatory markers to search for predictors of toxicity.

In an early analysis, this prephase strategy diminished treatment-related mortality while maintaining the expected survival rates. "We had a single event in 33 patients and no tumor lysis syndrome," he said. "This clinical maneuver allows 80% of patients to get through the treatment program."

Dose reductions are another way to minimize toxicity. With contemporary chemoimmunotherapy, curative outcomes (i.e., survival rates around 60% at 2 years) can still be maintained with reduced-intensity protocols, added Dr. Hamlin. "You can back off these regimens and make them more tolerable, and I think you can recapture that curative intent," he commented.

For the treatment of relapsed or refractory disease, the "mainstay of treatment," stem cell transplant, is too toxic for many older patients. Efforts are underway to modulate conditioning regimens to make transplant easier on these patients, but for most of them, transplant is not an option. For second-line regimens, a number of regimens can put patients into remission, yet currently one cannot be recommended over another, Dr. Hamlin admitted.

He acknowledged that since more and more patients with DLBCL are being cured with contemporary therapies, those who relapse tend to have poor biologic features, which make future responses less likely. "At the end of the day," noted Dr. Hamlin, "the impact of transplant has lessened."

## WHAT’S AHEAD: TARGETED THERAPY

The GCB subtype is associated with overexpression of genes involved in germinal center differentiation, such as *EZH2* and *BCL6*, whereas the ABC subtype is associated with chronic active B-cell receptor signaling and the constitutive activation of the nuclear factor kappa B pathway. Based on these differences, agents that selectively target components of these pathways may be beneficial in one but not both subtypes.

There are ongoing efforts to overcome the negative impact of the non-GCB subtype biology, using bortezomib (Velcade), idelalisib (Zydelig), lenalidomide (Revlimid), and ibrutinib (Imbruvica). Other potential targeted agents in non-GCB disease appear to be enzastaurin, fostamatinib, and the BCL2 inhibitor venetoclax (Venclexta); prospective studies are asking whether the addition of these agents to an R-CHOP backbone adds benefit.

Early data from a study by Nowakowski et al. (2015) found the addition of lenalidomide to R-CHOP improved progression-free survival in the non-GCB subtype, essentially negating its otherwise poor outcomes. This encouraging signal led to the randomized E1412 trial, which is being conducted by the Eastern Cooperative Oncology Group.

Interestingly, chemotherapy regimens may also have differential effects depending on cell of origin, with suggestions that DA-R-EPOCH is particularly effective in GCB, and that R-CHOP-ICE (ifosfamide, carboplatin, etoposide), and R-ACVBP (dose-intensified rituximab, doxorubicin, cyclophosphamide, vindesine, bleomycin, prednisone) may ameliorate non-GCB outcomes.

In the relapsed setting, Memorial Sloan Kettering researchers are evaluating salvage therapy according to the cell of origin. Transplant-eligible patients will receive R-ICE, with the addition of ibrutinib (to address the B-cell pathway) if they have the non-GCB/ABC subtype, and the antibody-drug conjugate SGN19a (which targets CD19) if they have the GCB subtype.

Other innovative approaches are the triplet of romidepsin (Istodax)/lenalidomide/carfilzomib (Kyprolis); the small-molecule inhibitors Syk/Jak, PI3K alone and PI3K plus Bruton’s tyrosine kinase inhibitors; the antibody-drug conjugate CD20-Shiga toxin MT3724; bispecific monoclonal antibodies; chimeric antigen receptor (CAR) T-cell therapy; and inhibitors of programmed cell death protein 1 and its ligand (PD-1/PD-L1).

"These approaches are still only in the realm of clinical research, but they are exciting," Dr. Hamlin concluded.
